# Klotho, an anti-aging gene, acts as a tumor suppressor and inhibitor of IGF-1R signaling in diffuse large B cell lymphoma

**DOI:** 10.1186/s13045-017-0391-5

**Published:** 2017-02-02

**Authors:** Xiangxiang Zhou, Xiaosheng Fang, Yujie Jiang, Lingyun Geng, Xinyu Li, Ying Li, Kang Lu, Peipei Li, Xiao Lv, Xin Wang

**Affiliations:** 1 Department of Hematology, Shandong Provincial Hospital affiliated to Shandong University, No.324, Jingwu Road, Jinan, Shandong 250021 People’s Republic of China; 2 Shandong University School of Medicine, Jinan, Shandong 250012 People’s Republic of China

**Keywords:** Diffuse large B cell lymphoma, Klotho, Insulin growth factor-1 receptor, Tumor suppressor

## Abstract

**Background:**

Klotho, is a transmembrane protein, performs as a circulating hormone and upstream modulator of the insulin-like growth factor-1 receptor (IGF-1R), fibroblast growth factor (FGF), and Wnt signaling pathways. These pathways are involved in the development and progression of B cell lymphoma. We aimed to explore the expression pattern and functional mechanism of Klotho in diffuse large B cell lymphoma (DLBCL).

**Methods:**

Immunohistochemistry (IHC) and western blotting were performed to detect the expression level of Klotho in DLBCL patients and cell lines. Tumor suppressive effect of Klotho was determined by both in vitro and in vivo studies. Signaling pathway activity was assessed by western blotting.

**Results:**

Remarkable lower expression levels of Klotho were observed in DLBCL patients and cell lines. Enforced expression of Klotho could significantly induce cell apoptosis and inhibit tumor growth in DLBCL. Upregulation of Klotho resulted in declined activation of IGF-1R signaling, accompanied with decreased phosphorylation of its downstream targets, including AKT and ERK1/2. Moreover, xenograft model treated with either Klotho overexpression vector or recombinant human Klotho administration presented restrained tumor growth and lower Ki67 staining.

**Conclusions:**

Our findings establish that Klotho performs as a tumor suppressor and modulator of IGF-1R signaling in DLBCL. Targeting Klotho may provide novel strategies for future therapeutic intervention.

## Background

Diffuse large B cell lymphoma (DLBCL) is the most common form of non-Hodgkin lymphoma (NHL), accounts for nearly 40% of all newly diagnosed cases [1]. This disease presents as an aggressive process and exhibits high heterogeneity in gene expression and clinical outcomes [2, 3]. Although majority of DLBCL patients could be cured by anthracycline-based chemotherapies combined with rituximab, one third of them presented refractory or relapsed process [4, 5]. Therefore, more effective treatment strategies based on novel therapeutic targets and molecular oncogenic pathways are still needed.

Klotho is an anti-aging gene originally identified in 1997 [6]. Kuro-o et al. [6, 7] found that Klotho-deficient mice developed multiple premature aging syndromes, whereas overexpression of Klotho extended the lifespan of mice. The Klotho gene is located in chromosome 13q12 in human with the size of 50 kb [6]. It encodes a single-pass transmembrane protein, which consists of an extracellular domain, a single transmembrane domain, and an intracellular domain. The intracellular domain is very short and has no clear functions. Membrane Klotho functions as an obligate co-receptor of fibroblast growth factor 23 (FGF23) to regulate phosphate homeostasis [8]. The extracellular domain (secreted Klotho) could be released into the serum and functions as a circulating hormone to regulate the activity of oxidative stress, multiple growth factor receptors, and ion channels [9, 10].

The tumor suppressive activity of Klotho was first identified in breast cancer in 2008 [11]. Recent investigations have implicated that Klotho is extensively downregulated in several solid tumors, including cervical cancer, pancreatic cancer, melanoma, and several digestive neoplasm [12]. In these malignancies, Klotho was elucidated to be a modulator of several signaling pathways, including the FGF signaling, insulin-like growth factor-1 receptor (IGF-1R), and Wnt pathways, which are also involved in the pathogenesis of hematological malignancies [10, 13–15]. However, the role of Klotho in hematological malignancies has not been reported.

A large number of aberrant receptor tyrosine kinases (RTKs) have been found in hematological malignancies [16, 17], but they are still indefinite in DLBCL. IGF-1R is a RTK primarily activated by its cognate ligands, IGF-1, and IGF-2. It plays a crucial role in the establishment and progression of tumors by regulating proliferation, self-renewal, apoptosis, and drug resistance of cancer cells [18–20]. Activation of IGF-1R following IGF-1 treatment results in phosphorylation of downstream signaling cascades, including PI3K/AKT and MAPK/ERK [19]. Blockade of PI3K/AKT signaling could restrain cell survival and function of lymphocytes [21, 22]. Activation of MAPK/ERK signaling promotes cell proliferation and metastasis of cancer cells [23]. Strategies to block IGF-1R pathway in solid malignancies are being tested in clinical trials [24], whereas the function of IGF-1R signaling in DLBCL has been less studied [25].

In this present study, we aimed to assess the expression level and functional mechanism of Klotho in DLBCL. We identified reduced expression of Klotho in DLBCL for the first time. Noted inhibition of cell growth and induction of apoptosis were observed in DLBCL with Klotho overexpression. Tumor growth was restrained by administration of Klotho protein in xenograft model of DLBCL. Our findings demonstrated that Klotho was a tumor suppressor and modulator of IGF-1R signaling in DLBCL, indicating that targeting Klotho may provide novel therapeutic strategy in DLBCL.

## Methods

### Patients

This study was approved by the Medical Ethical Committee of Shandong Provincial Hospital affiliated to Shandong University. The paraffin-embedded archived samples from 50 newly diagnosed DLBCL patients and 20 reactive hyperplasia patients were collected. Samples of patients with reactive hyperplasia were referred as control. Histological diagnoses were established according to the WHO classification [26]. Peripheral blood mononuclear cells (PBMCs) from the whole blood of healthy donors were isolated using Ficoll-Hypaque density gradient centrifugation (TBD science, Tianjin, China). Normal peripheral blood CD19+ B cells were purified from freshly isolated PBMCs using CD19+ magnetic microbeads kit (Miltenyi Biotec, Bergisch Gladbach, Germany). Cells were incubated with beads for 15 min at 4 °C while rotating. Purified CD19+ B cells were selected according to the manufacturer’s protocols. The purity of isolated populations was assessed by FACS analysis, and cells with >90% purity were collected. All samples were obtained with informed consent in accordance with the Declaration of Helsinki.

### Cell lines and reagents

Human DLBCL cell lines LY1 and LY8 were cultured in Iscove modified Dulbecco medium with 10% heat-inactivated fetal bovine serum. The medium contains 1% penicillin/streptomycin mixture and 2 mmol/l glutamine. CD19+ B cells and PBMCs obtained from three healthy donors were used as controls (N1, N2, and N3 cells). Recombinant human Klotho (rhKL) and recombinant human IGF-1 were obtained from PeproTech (Rocky Hill, NJ, USA), and adriamycin (ADR) was bought from Actavis (S.p.A, Italy).

### Cell transfection

Lentivirus vectors either encoding Klotho or an empty lentiviral vector were from Genechem (Shanghai, China). Lentivirus transfection was carried out according to the manufacturers’ instruction. Infection efficiencies were assessed by green fluorescent protein (GFP) through flow cytometry. The stably transfected cells were selected 48 h later with 5 μg/ml puromycin (Sigma-Aldrich, USA).

### Quantitative real-time PCR

Total RNA was extracted using RNAiso Plus (TaKaRa, Dalian, China). Reverse transcription reaction was conducted with the reverse transcription reagents (TaKaRa, Dalian, China). Amplification reactions were performed with SyberGreen (TaKaRa, Dalian, China) in LightCycler 480II (Roche, Basel, Swizerland). Klotho-specific primers were as follows: forward, 5′-AGCAATCTGGTCTGAATAACACTGG; reverse, 5′-CATGTTTCAGCGTGAAAGTTCAAAG. Relative quantification was calculated using the ^△△^CT method.

### Immunohistochemistry (IHC) and hematoxylin-eosin staining

The 4-μm-thick formalin-fixed, paraffin-embedded tissue sections were deparaffinized and hydrate. Antigen retrieval was performed using 0.01 mol/l sodium citrate buffer (pH 6.0) under high pressure followed by a 1-h cool-down and rinses in phosphate buffer solution (PBS). Endogenous peroxidase was blocked with 3% hydrogen peroxide in methanol for 15 min, followed by incubation with normal serum to block non-specific binding. The slides were then incubated overnight at 4 °C with primary antibodies, anti-Klotho (1:150) or anti-Ki67 (1:100). After washing, the tissue sections were treated with the second antibody from SP reagent kit (Zhongshan Goldenbridge, Beijing, China) for 30 min at room temperature, followed by further treated with strept avidin-horseradish peroxidase complex (SABC) for 30 min at room temperature. After treated with diaminobenzidine (DAB) Kit (ZhongshanGoldenbridge, Beijing, China), the stained slides were counterstained with hematoxylin and mounted. Negative control was carried out with the primary antibody replaced by PBS. IHC staining was scored by the proportion of positive tumor cells. Five microscopic fields with the highest immunoreactivity at ×400 magnification were evaluated by two independent observers who were blinded to the patients’ clinical data. Cases with at least 10% of tumor cells with Klotho staining were considered as positive. Fresh mice subcutaneous tumors were fixed in 4% paraformaldehyde (PFA) and embedded with paraffin for histological examinations. Sections with 4-μm thickness were cut and stained with hematoxylin-eosin (H&E).

### Western blotting

Cells were lysed in radio-immunoprecipitation assay buffer (Shenergy Biocolor, Shanghai, China) together with 1× phosphatase inhibitor cocktail (PhosSTOP; Roche, Mannheim, Germany). The BCA assay (Shenergy Biocolor, Shanghai, China) was performed to detect protein concentration. Protein extracts (30 μg) were then electrophoresed on SDS-polyacrylamide gels and blotted from the gel onto polyvinylidene fluoride membranes (Millipore, Billerica, MA, USA). Membranes were incubated with the blocking solution (5% skim milk in Tris-buffered saline containing 0.05% Tween-20) for 1 h at room temperature and then immunoblotted with the indicated antibodies (1:1000 dilution) overnight at 4 °C. After which, the membranes were washed with TBS-T and then probed with the HRP-conjugated secondary antibodies (Zhongshan Goldenbridge, Beijing, China) and the electro-chemi-luminescence kit (Millipore, Billerica, MA, USA). Chemiluminescent signals were detected using the Amersham Imager 600 imaging system (General Electric, USA). ImageJ software (ImageJ, NIH) was used to quantify the protein bands normalized to control. Primary antibodies used were Klotho (Abcam, Cambridge, MA, USA), phospho-IGF-1R (Tyr1135/1136), IGF-1R, phospho-AKT(Ser473), total pan-AKT, Mcl-1, Caspase-3, diphosphorylated and total ERK1/2 (Cell Signaling Technologies, Beverly, MA, USA), β-actin, and GAPDH (Zhongshan Goldenbridge, Beijing, China). The experiments were performed in triplicate with GAPDH or β-actin (Zhongshan Goldenbridge, Beijing, China) as endogenous control.

### Cell proliferation assay

Cell proliferation was assessed by performing triplicate assays with the Cell Counting Kit-8 (CCK-8) assay (Enogene, Nanjing, China). DLBCL cells with designed treatment were seeded in 96-well plates at a density of 5000 cells/well for 48 or 24~96 h later. Thereafter, the cells were incubated with 10 μl/well CCK-8 for 4 h according to the manufacturer’s proposal. Cell proliferation was detected by light absorption at 450 nm by Multiskan GO Microplate Reader (Thermo Scientific, Rockford, IL, USA).

### Flow cytometry analysis

Apoptosis of transfected DLBCL cells were detected by Annexin V-PE/7-aminoactinomycin (7AAD) (BD Biosciences, Bedford, MA, USA) assay according to the manufacturers’ instructions. DLBCL cells with designed treatments were harvested and washed twice with ice-cold PBS and incubated in 1× binding buffer (containing 5 μl Annexin V-PE and 5 μl 7AAD). After incubation in the dark for 15 min, cells were subjected to the flow cytometry. At least 10,000 events per sample were acquired. Cells were discriminated into viable cells (AnnexinV-PE^−^/7AAD^−^), dead cells (Annexin V-PE^−^/7AAD^+^), apoptotic cells (Annexin V-PE^+^/7AAD^−^), and necrotic cells (Annexin V-PE^+^/7AAD^+^). The rates of apoptotic cells were acquired on a FACS-Navios Flow Cytometer (Beckman Coulter, CA, USA). Data were analyzed using FlowJo Version 7.6 software (Tree Star Inc., OR, USA).

### Elisa assay

Serum soluble Klotho levels were measured using the Elisa kit (Immuno-Biological Laboratories, Gunma, Japan), with a lower limit of assay of 6.15 pg/ml.

### In vivo xenograft study

All animal experiments were performed in accordance with the principles of the Institutional Animal Care. Severe combined immunodeficiency (SCID) Beige female mice of 5-week old were bought (Weitong Lihua Laboratory Animal Center, Beijing, China) and maintained in a pathogen-free environment under controlled condition of light and humidity. 1 × 10^7^ LY1 cells (untransfected, stably Klotho-overexpressing vector tranfected, or empty control vector transfected, respectively), mixed with 100 μl Matrigel (BD Biosciences, Bedford, MA, USA), were subcutaneously injected into the right inferior limb of mice. Tumor size was measured by the digital caliper. For the experiment with rhKL, SCID Beige mice were injected subcutaneously with 1 × 10^7^ LY1 cells into the left inferior limbs. Mice were treated with daily intraperitoneal injections of rhKL (7.5 μg/kg) or PBS control (six mice per group) for 2 weeks. The volume of tumor was estimated using the equation *V* = (*a* × *b* ^2^) × 0.5236, where *a* is the largest dimension and *b* is the perpendicular diameter.

### Statistical analysis

All statistical analyses were performed by using the statistic software SPSS17.0 (SPSS Inc., Chicago, IL, USA) for Windows. In vitro experimental results were presented as mean ± SD of data obtained from three separate experiments. Overall survival time was measured from the date of diagnosis to the date of death or the last follow-up. Kaplan-Meier analysis was performed to estimate the survival functions. A log-rank test was used to assess survival differences. Chi-square test was used to analyze the clinical characteristics of patients. One-way analysis of variance (ANOVA) or *t* tests were used to assess the differences between groups. *p* < 0.05 was considered to be statistically significant.

## Results

### Klotho was downregulated in DLBCL and related to tumor progression

**Fig. 1:**
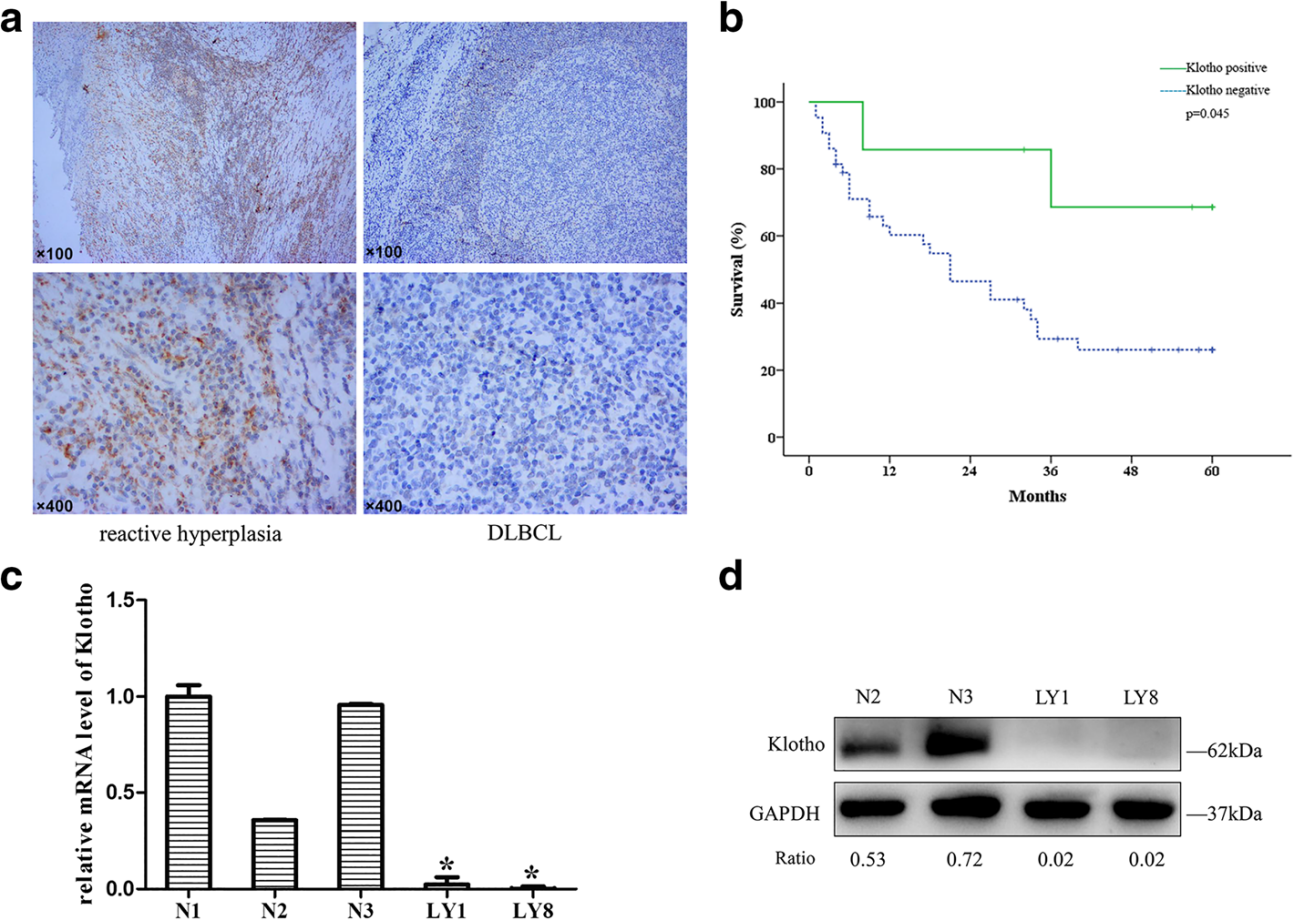
Klotho was downregulated in DLBCL and related to tumor progression. **a** Compared with reactive hyperplasia, expression level of Klotho was significantly decreased in DLBCL tissues. Original magnification, ×100 (*upper panel*) and ×400 (*lower panel*). **b** Patients with Klotho negative expression showed shorter survival than those with Klotho positive expression. **c** As detected by real-time quantitative PCR, lower levels of Klotho mRNA expression were observed in DLBCL cell lines (LY1, LY8) than in CD19+ B cells (N1, N2, N3) (mean ± SD, *n* = 3, *p < 0.05). **d** Protein expression levels of Klotho were detected in DLBCL cells and normal PBMCs. The ratios of relative protein expression level of targets are indicated below the western blot

Compared with reactive hyperplasia, expression level of Klotho was significantly lower in DLBCL tissues (Fig. 1a). Klotho positive rate was 14% (7 of 50) in DLBCL whereas 80% (16 of 20) in reactive hyperplasia. To evaluate the clinical significance of Klotho in DLBCL, clinical and pathological characteristics of DLBCL patients were analyzed. The expression level of Klotho was associated with the Ann Arbor stage of DLBCL patients. Patients with stage III or IV presented markedly lower Klotho level than those with stage I or II (*p* = 0.002, Table 1). In addition, Kaplan-Meier survival analysis were performed based on the expression levels of Klotho (*n* = 50). The median survival of patients in Klotho-positive group was 48.46 months, significantly longer than those in Klotho-negative group (29.27 months, *p* = 0.045, Fig. 1b). We next confirmed the expression of Klotho in DLBCL cell lines. Klotho mRNA expression was detected in DLBCL cell lines and normal CD19+ B cells. Significantly decreased levels of Klotho mRNA expression were observed in DLBCL cell lines (LY1 0.024 ± 0.037, LY8 0.002 ± 0001, Fig. 1c). Reduced expression levels of Klotho protein were also noted in DLBCL cell lines compared to human PBMCs (Fig. 1d).

**Table 1:** Correlation between Klotho expression and clinical characteristics of DLBCL patients

Characteristics	No. of patients	Klotho expression		*p* value
		Positive (%)	Negative	
Age (years)				
<60	27	4(14.8%)	23	0.857
≥60	23	3(13.0%)	20	
Gender				
Male	26	2(7.7%)	24	0.181
Female	24	5(20.8%)	19	
Ann Arbor stage				
I or II	12	5(41.7%)	7	0.002
III or IV	38	2(5.3%)	36	
B symptoms				
Present	14	2(14.3%)	12	0.971
Absent	36	5(13.9%)	31	
Subtype				
GCB	15	2(13.3%)	13	0.929
Non-GCB	35	5(14.3%)	30	
Serum LDH				
Normal	35	5(14.3%)	30	0.929
Elevated	15	2(13.3%)	13	
Extranodal involvement				
Absent	28	4(14.3%)	18	0.45
Present	22	3(13.7%)	25	
IPI score				
0–2	35	6(17.1%)	29	0.328
3–5	15	1(6.7%)	14	

*LDH* lactate dehydrogenase, *IPI* international prognostic index

### Klotho inhibited growth of DLBCL

**Fig. 2:**
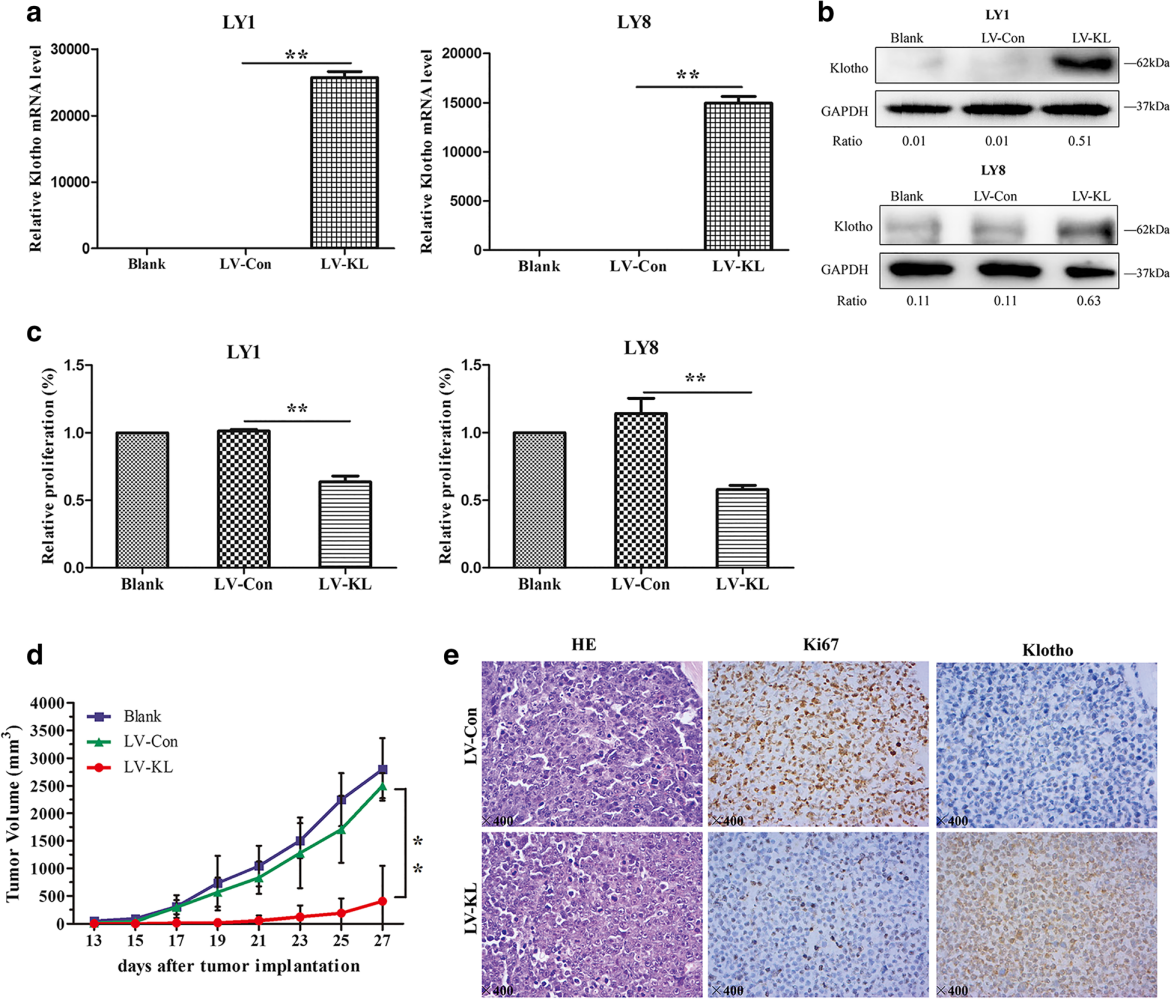
Klotho suppressed DLBCL growth. **a**, **b** Relative expression levels of Klotho were confirmed by quantitative PCR (mean ± SD, *n* = 3, **p < 0.01) and western blot in stably transfected LY1 and LY8 cells compared to empty vectors. The ratios of relative protein expression level of targets are indicated below the bands. **c** DLBCL cells transfected with LV-KL presented significantly lower level of cell proliferation than those transfected with empty vector (mean ± SD, *n* = 3, **p < 0.01). **d** SCID mice with Klotho overexpression revealed significantly lower tumor volume than those with empty vector (*n* = 6 per group, **p < 0.01). **e** H&E staining and IHC staining of Ki67 and Klotho were performed in mice tumors. Original magnification, ×400

To further establish the biological function of Klotho, human DLBCL cells were stably transfected with either negative control lentivirus vectors (LV-Con) or Klotho-overexpression lentivirus vectors (LV-KL). The upregulation of Klotho levels were confirmed by quantitative PCR and western blot (Fig. 2a, b). Cell proliferation rates were evaluated by CCK-8 assay. Significant reduction of cell proliferation was observed in LY1 and LY8 cells transfected with LV-KL, compared with those transfected with empty vector (Fig. 2c). Furthermore, we investigated the anti-tumor effect of Klotho on DLBCL xenografts in vivo. LY1 cells, stably expressing Klotho, or the vector control cells were injected subcutaneously into SCID Beige mice, respectively. Consistent with the in vitro results, mice treated with Klotho overexpression cells revealed remarkable reduction in tumor volume compared to that transfected with empty vector (Fig. 2d). Higher expression level of Klotho was identified in mice treated with LV-KL-transfected LY1 cells (Fig. 2e). In addition, we estimated the expression level of proliferative marker Ki67 [27, 28] in xenograft mice by IHC. Lower level of Ki67 positive rate were observed in LV-KL group (Fig. 2e).

### Klotho promoted apoptosis of DLBCL cells

**Fig. 3:**
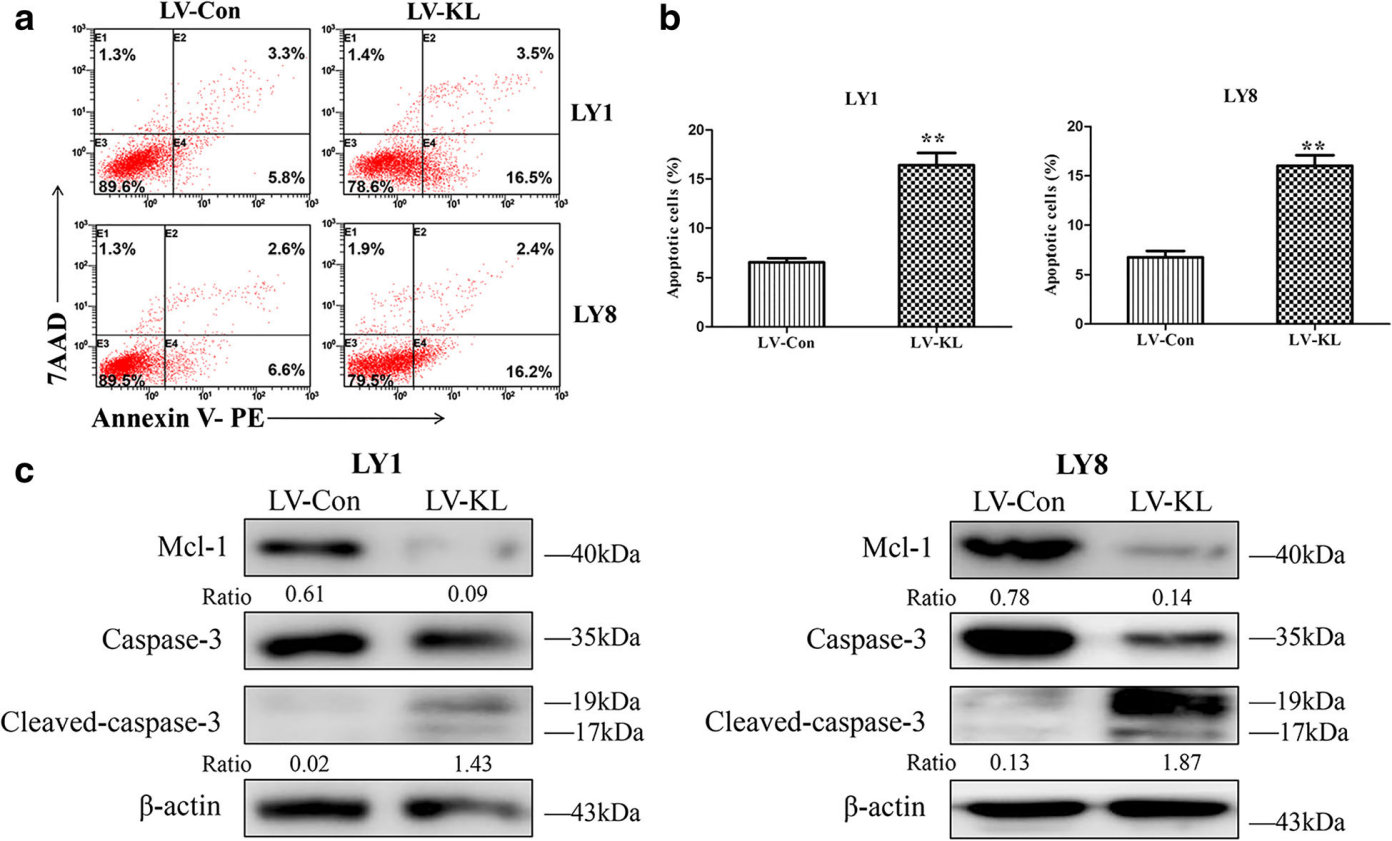
Klotho promoted apoptosis of DLBCL. **a**, **b** Enforced expression of Klotho resulted in increased apoptosis rates in LY1 and LY8 cells assessed by flow cytometric analysis with Annexin V-PE/7AAD staining (mean ± SD, *n* = 3, **p < 0.01). **c** Declined expression levels of Mcl-1 and increased levels of activated Caspase-3 were observed in LV-KL-treated DLBCL cells. The ratios of relative protein expression level of targets are indicated below the bands

To investigate whether Klotho could promote the apoptosis of DLBCL cells, Annexin-V-based apoptotic assays were performed. Flow cytometry analysis indicated that Klotho overexpression resulted in enforced apoptosis rates in both LY1 (6.56 ± 0.71% in LV-Con vs. 16.41 ± 2.16% in LV-KL group, *p* = 0.002) and LY8 cells (7.23 ± 0.65% in LV-Con vs. 16.03 ± 1.85% in LV-KL group, *p* = 0.001, Fig. 3a, b). In addition, western blot analysis confirmed the pro-apoptotic effects of Klotho. Dramatically reduction of anti-apoptotic protein Mcl-1 and increased cleaved forms of Caspase-3 were observed in DLBCL cell lines (Fig. 3c). Altogether, these results indicate that upregulation of Klotho could promote the apoptosis of DLBCL cells.

### Klotho modulated the activation of IGF-1R signaling in DLBCL

**Fig. 4:**
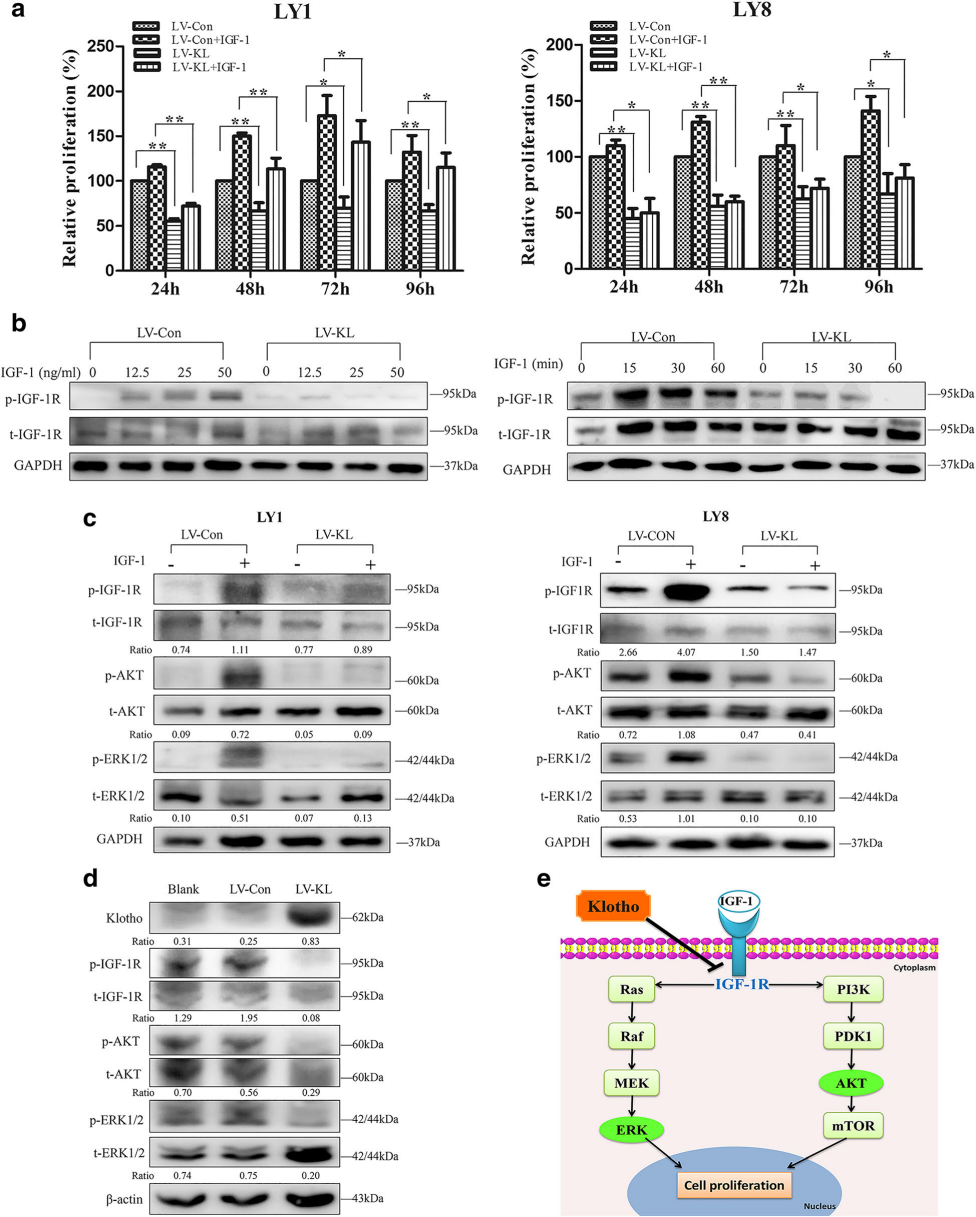
Klotho modulated activation of IGF-1R pathway in DLBCL. **a** IGF-1-induced DLBCL cell (LY1 and LY8) proliferation is inhibited by Klotho overexpression. LY1 and LY8 cells were transfected with LV-KL or LV-Con, starved for 48 h and treated by IGF-1 (50 ng/ml) and analyzed by CCK-8 assay (mean ± SD, *n* = 3, *p < 0.05, **p < 0.01). **b** LY1 with stable transfection of LV-KL or LV-Con were serum starved for 48 h and treated with IGF-1 (50 ng/ml) for the indicated times or IGF-1 (30 min) for the indicated doses. After treatment, cells were harvested and analyzed by western blot. **c** LY1 and LY8 cells transfected with LV-KL or LV-Con, serum starved for 48 h, and treated with IGF-1 (50 ng/ml, 30 min). Western blot was conducted to assess the phosphorylated (p) and total (t) protein levels of IGF-1R, AKT, and ERK1/2. The ratios of relative protein expression level of targets are indicated below the bands. **d** Decreased activation of IGF1-R signaling was observed in LV-KL-treated mice. The ratios of relative protein expression level of targets are indicated below the bands. **e** Schematic description of Klotho mediated IGF1R signaling

Having shown that Klotho could impair cell proliferation and induce apoptosis in DLBCL, we next investigated the molecular mechanisms responsible for the function of Klotho. The IGF-1R pathway plays a vital role in the development of hematological malignancies [14, 25, 29]. CCK-8 assay was conducted to assess the effect of Klotho on IGF-1-induced cell proliferation. DLBCL cells transfected with either LV-KL or LV-Con were treated with IGF-1 or vehicle control in 0.5% FBS culture medium for 24–96 h. In the groups untreated with IGF-1, LV-KL transfection resulted in declined proliferation of LY1 and LY8 cells compared to empty-vector group. In the IGF-1-treated groups, we observed that cell proliferation was less restored by IGF-1 in cells transfected with LV-KL compared to that transfected with LV-Con. Following addition of IGF-1, cell proliferation of LV-Con-treated cells increased by up to 60%, whereas the only up to 40% enhancement of cell proliferation was found in LV-KL transfected cells (Fig. 4a).

**Fig. 5:**
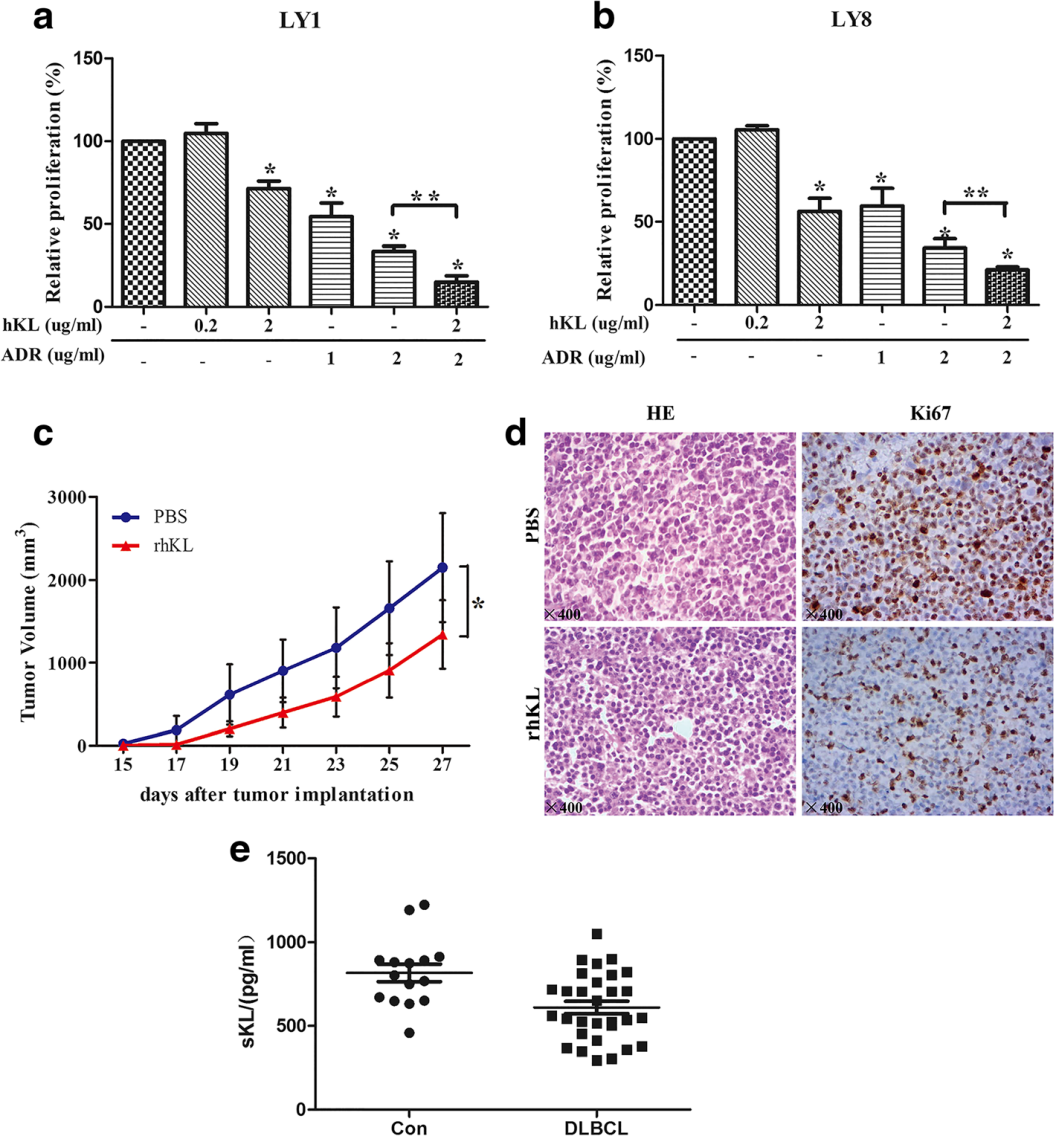
rhKL acted as an active form in vitro and vivo. **a**, **b** LY1 and LY8 cells were treated with rhKL, ADR, their combination, or a vehicle control. CCK-8 assay was conducted after 48 h (mean ± SD, *n* = 3, *p < 0.05 for comparison between treated cells and control, **p < 0.05 between Klotho and ADR combination versus ADR alone). **c** LY1 cells were injected subcutaneously into the left inferior legs of SCID Beige mice. The mice were treated with daily intraperitoneal injections of rhKL (7.5 μg/kg) or vehicle control (PBS) for 2 weeks. Tumor volumes were measured every 2 days (*n* = 6 per group, *p < 0.05). **d** H&E staining and IHC staining with Ki67 were performed. Original magnification, ×400. **e** Lower serum Klotho levels were detected by Elisa in DLBCL patients than the control subjects

As the IGF-1R signaling could be activated by IGF-1, we explored the optimal activation dose and time of IGF-1 in DLBCL cell lines. LY1 cells were transfected with either LV-KL or LV-Con, serum starved for 48 h and treated with IGF-1 for the indicated times and doses. Western blot was carried out to evaluate the phosphorylation level of the IGF-1R. As shown in Fig. 4b, the maximum activation occurred at IGF-1 concentration of 50 ng/ml and at the time of 30 min after treatment. Then, we studied the ability of Klotho to modulate activation of IGF-1R signaling in DLBCL cells. Cells transfected with either LV-KL or LV-Con were serum starved for 48 h, treated with IGF-1 (50 ng/ml for 30 min) or vehicle control. Following treatment, cells were harvested and immunoblotting was conducted. Decreased phosphorylation level of IGF-1R and its downstream targets, including AKT and ERK1/2, were observed in cells transfected with LV-KL (Fig. 4c). Furthermore, we evaluated the modulation of Klotho on IGF-1R signaling in DLBCL xenograft model*.* Enhanced expression of Klotho in LV-KL-treated mice was confirmed by immunoblotting (Fig. 5d). Decreased phosphorylation of IGF1-R as well as its downstream targets were observed in mice treated with LV-KL compared to the control group (Fig. 4d). These results demonstrated that Klotho may act as a modulator of IGF-1R signaling contributed to the tumorigenesis of DLBCL (Fig. 4e).

### rhKL acted as an active form both in vitro and in vivo

We further investigated the activity of secreted Klotho protein on DLBCL. To explore the effect of secreted Klotho on proliferation of DLBCL cells, LY1 and LY8 cells were seeded and treated with rhKL. The rhKL was found to be active and reduced the proliferative ability of DLBCL cells by 30~40% at the concentration of 2 μg/ml (Fig. 5a, b). ADR is one of the most commonly used drugs in the chemotherapeutic strategy of DLBCL. We detected the function of Klotho on cell responses to ADR. LY1 and LY8 cells were treated for 48 h with rhKL, ADR, or their combination, and cell proliferation was then evaluated. Combination of rhKL (2 μg/ml) increased the sensitivity of DLBCL cells to ADR (Fig. 5a, b).

The in vivo activity of rhKL in DLBCL xenograft model was also detected. LY1 cells were injected subcutaneously into the SCID Beige mice to build a DLBCL xenograft model. After 15 days, the mice were treated with daily intraperitoneal injection of either rhKL (7.5 μg/kg, *n* = 6) or a control vehicle (*n* = 6) for 2 weeks. Significant decreased tumor volumes were observed in the mice treated with rhKL compared with those treated with vehicle control (Fig. 5c). Moreover, reductive expression level of proliferative marker Ki67 was found in rhKL-treated mice (Fig. 5d).

In addition, we employed ELISA assays to test the soluble Klotho levels in the serum of 30 initially diagnosed DLBCL patients and 15 healthy volunteers. Lower serum Klotho levels were noted in DLBCL patients (628.54 ± 219.39 pg/ml) than the control subjects (818.87 ± 241.51 pg/ml, *p* = 0.045, Fig. 5e).

## Discussion

In this study, our observations identified for the first time that Klotho, an anti-aging gene, as a potential tumor suppressor in DLBCL tumorigenesis. Klotho was downregulated in human DLBCL and inhibited the growth of DLBCL both in vitro and in vivo. Upregulation of Klotho resulted in declined activation of IGF-1R signaling pathway in DLBCL.

We identified remarkable reduced expression of Klotho in DLBCL tissues and cell lines, whereas higher expression in reactive hyperplasia and CD19+ B cells from normal donors. Decreased level of Klotho was associated with advanced stage and more aggressive disease process in DLBCL. Tumor suppressive activity of Klotho has been reported in several human solid malignancies, but never in hematological cancers [30–33]. Recent investigations elucidated that epigenetic mechanisms, including promoter methylation and histone deacetylation, contributed to the reduced Klotho expression in human breast cancer, cervical cancer, and hepatocellular carcinomas [34–36]. The similar mechanism may be involved in DLBCL. At present, the diagnosis of DLBCL is mainly based on the biopsy histopathology and IHC [37]. With the deepening of additional studies, lower expression of Klotho may serve as a potential marker for the pathological diagnosis of DLBCL.

DLBCL is a high-grade heterogeneous disorder defined by heterogeneity in clinical and biological characteristics [1, 38, 39]. Personalized prognostic stratification and targeted therapeutic strategies are urgently needed to improve the outcomes of DLBCL patient [40]. Ki67 as a proliferative marker performed as a poor prognostic marker in DLBCL [27]. In this study, we discovered that DLBCL xenograft mice with Klotho overexpression exhibited significantly lower Ki67 staining positive rate than that without Klotho upregulation.

As Klotho exists in both membrane-bound form and secreted form, the secreted Klotho could be shed and released into the circulation [7]. Recent investigation elucidated the low serum level of Klotho in renal cell carcinoma [41]. Low serum Klotho level was an independent adverse prognostic factor for cancer-specific and progression-free survival in RCC [41]. However, the level of serum soluble Klotho was unchanged in multiple myeloma [42]. Thus, the diagnostic role of secreted Klotho in human cancer remains controversial. In this study, we identified the decreased level of serum Klotho in DLBCL patients. The correlation of serum Klotho with disease diagnosis and progression still needs further exploration. Larger number of the included patients will better confirm the role of serum Klotho in DLBCL.

Importantly, our study illuminated that Klotho effectively inhibited the growth of DLBCL cells. Overexpression of Klotho significantly inhibited cell proliferation and induced cell apoptosis in DLBCL. We also discovered that efficacy of ADR could be enhanced by combination with rhKL in DLBCL cells. This finding suggested the potential of Klotho in therapeutic intervention of DLBCL. Our in vivo investigations demonstrated that upregulation of Klotho, either by LV-KL transfection or rhKL administration, congruously led to inhibitory effect in the tumor growth of xenograft model of DLBCL. This will pave the way for rational design of Klotho-based molecular products in DLBCL. Nevertheless, pharmacokinetic investigations are still required to explore the optimal dose and adverse reaction of rhKL in the use of either single agent treatment or drug combination.

Significantly, we also found that Klotho could inhibit the activation of IGF-1R signaling in DLBCL. Induction of IGF-1R signaling was involved in the pathogenesis of hematological malignancies [13]. Recently, Stromberg et al. reported the involvement of IGF-1R and the antitumor effects of specific IGF-1R inhibitors in DLBCL tumorigenesis [25]. Structure-function analysis of Klotho indicated that Klotho could interact with the IGF-1R [43]. Klotho-induced inhibition of IGF-1R signaling may act as a novel mechanism involved in the development of DLBCL. Apart from IGF-1R signaling, many other pathways have been confirmed to be modulated by Klotho in tumorigenesis. The most studied is the FGFR-Klotho axis, and Klotho could act as cofactor of endocrine FGFs to bind and induce the activation of FGFRs in breast, pancreatic, and prostate cancers [11, 30, 44]. Deregulation of Wnt signaling pathway plays a critical role in the pathogenesis of cancers [45–47]. It was proved that Klotho participated in tumorigenesis partly through restraining the Wnt signaling pathway [48]. The modulation of Klotho on these pathways in DLBCL is poorly understood. In spite of the important discoveries revealed by these studies, there are also limitations on the detailed mechanisms and the cross-talks of them involved in Klotho deregulation.

## Conclusions

Taken together, our findings identified that Klotho performs as tumor suppressor and modulator of IGF-1R signaling in the DLBCL. Overexpression of Klotho may be a predictive marker for favorable outcome in DLBCL. Klotho reinforces the response of DLBCL cells to chemotherapeutic drug. Being an endogenous circulating hormone, the secreted Klotho could function as an active form and inhibit the tumor growth effectively both in vitro and in vivo. This study illuminates Klotho as a potential target for future therapeutic strategies.

## Abbreviations

ADR: Adriamycin
CCK-8: Cell Counting Kit-8
DAB: Diaminobenzidine
DLBCL: Diffuse large B cell lymphoma
FGF23: Fibroblast growth factor 23
GFP: Green fluorescent protein
H&E: Hematoxylin-eosin
IGF-1R: Insulin-like growth factor-1 receptor
IHC: Immunohistochemistry
IPI: International prognostic index
LDH: Lactate dehydrogenase
LV-Con: Negative control lentivirus vectors
LV-KL: Klotho-overexpression lentivirus vectors
NHL: Non-Hodgkin lymphoma
PBMCs: Peripheral blood mononuclear cells
PBS: Phosphate buffer solution
PFA: Paraformaldehyde
rhKL: Recombinant human Klotho
RTKs: Receptor tyrosine kinases
SABC: Strept avidin-horseradish peroxidase complex
SCID: Severe combined immunodeficiency
